# Technologies for Innovative Monitoring to Reduce Blood Pressure and Change Lifestyle Using Mobile Phones in Adult and Elderly Populations (TIM Study): Protocol for a Randomized Controlled Trial

**DOI:** 10.2196/resprot.9619

**Published:** 2018-08-07

**Authors:** Sandra C Fuchs, Erno Harzheim, Cirano Iochpe, Caroline N de David, Marcelo R Gonçalves, Guilhermo P Sesin, Cassio M Costa, Leila B Moreira, Flavio D Fuchs

**Affiliations:** ^1^ Postgraduate Studies Program in Cardiology, Hospital de Clinicas de Porto Alegre School of Medicine Universidade Federal do Rio Grande do Sul Porto Alegre Brazil; ^2^ Postgraduate Studies Program in Epidemiology School of Medicine Universidade Federal do Rio Grande do Sul Porto Alegre Brazil; ^3^ Informatics Institute Universidade Federal do Rio Grande do Sul Porto Alegre Brazil; ^4^ TelessaúdeRS School of Medicine Universidade Federal do Rio Grande do Sul Porto Alegre Brazil; ^5^ Division of Cardiology Hospital de Clinicas de Porto Alegre Universidade Federal do Rio Grande do Sul Porto Alegre Brazil

**Keywords:** blood pressure, blood pressure monitoring, hypertension, weight, diet, sodium, physical activity, randomized controlled trial, text messages

## Abstract

**Background:**

Hypertension is a growing problem worldwide, markedly in low- and middle-income countries, where the rate of control slightly decreased. The overall prevalence of hypertension in Brazil is 28.7% among adult individuals and 68.9% in the population aged 60 years and older, and less than a third of patients have controlled blood pressure (BP). The use of technologies—mobile phones and the internet—to implement interventions to reduce blood pressure can minimize costs and diminish cardiovascular risk. Interventions through text messaging and electronic BP monitoring present divergent results.

**Objective:**

This trial evaluates the effectiveness of interventions—personalized messages and telemonitoring of BP—to reduce systolic BP and improve lifestyle compared to the usual care of patients with hypertension (control group).

**Methods:**

This factorial randomized controlled trial enrolls individuals aged 30 to 75 years who have a mobile phone and internet access with the diagnosis of hypertension under drug treatment with up to 2 medications and uncontrolled BP. Eligible participants should have both increased office BP and 24-hour BP with ambulatory BP monitoring. Participants with severe hypertension (systolic BP ≥180 or diastolic BP ≥110 mm Hg), life threatening conditions, low life expectancy, recent major cardiovascular event (last 6 months), other indications for the use of antihypertensive medication, diagnosis of secondary hypertension, pregnant or lactating women, or those unable to understand the interventions are excluded. Participants are randomly allocate to 1 of 4 experimental arms: (1) Telemonitoring of blood pressure (TELEM) group: receives an automatic oscillometric device to measure BP, (2) telemonitoring by text message (TELEMEV) group: receives personalized, standardized text messages to stimulate lifestyle changes and adhere with BP-lowering medication, (3) TELEM-TELEMEV group: receives both interventions, and (4) control group: receives usual clinical treatment (UCT). Data collection is performed in a clinical research center located in a referent hospital. The primary outcomes are reduction of systolic BP assessed by 24-hour ambulatory BP monitoring (primary outcome) and change of lifestyle (based on dietary approaches to stop hypertension (DASH)-type diet, sodium restriction, weight loss or control, increase of physical activity).

**Results:**

This study was funded by two Brazilian agencies: the National Council for Scientific and Technological Development and Fundação de Amparo à Pesquisa do Estado do Rio Grande do Sul. Enrollment was completed at the end of 2017 (N=231), the follow-up is ongoing, and data analysis is expected to begin in early 2019. A reduction of 24-hour systolic BP of approximately 8.8 [SD 13.1] mm Hg for participants in the BP monitoring group versus 3.4 [SD 11.6] mm Hg in the UCT group is expected. A similar reduction in the text messaging group is expected.

**Conclusions:**

The use of mobile technologies connected to the internet through mobile phones promotes time optimization, cost reduction, and better use of public health resources. However, it has not been established whether simple interventions such as text messaging are superior to electronic BP monitoring and whether both outperform conventional counseling.

**Trial Registration:**

ClinicalTrials.gov NCT03005470; https://clinicaltrials.gov/ct2/show/NCT03005470 (Archived by WebCite at http://www.webcitation.org/70AoANESu). Plataforma Brasil CAAE 31423214.0.0000.5327.

**Registered Report Identifier:**

RR1-10.2196/9619

## Introduction

Hypertension is a growing problem worldwide, markedly in low- and middle-income countries where increased prevalence was not followed up by higher awareness and control rates [1]. The overall prevalence of hypertension in Brazil is 28.7% (95% CI 26.2% to 31.4%) among adults [2] and 68.9% (95% CI 64.1% to 73.3%) in the population aged 60 years and older [3], and less than a third of patients have controlled blood pressure (BP). Low- and middle-income countries may even be facing a persistent increase of individuals with high BP in the next decade [1,4]. Successful attempts have been shown to increase BP control [5,6] but have not been implemented in clinical practice.

In recent years, the spectrum of interventions to increase hypertension control and reduce cardiovascular risk factors has widened as researchers seek alternatives that do not overburden the public health system [7]. The use of technologies using mobile phones and the internet to implement interventions can improve BP control, minimize health care resource use and costs [8], and reduce cardiovascular risk [7,9]. However, the effectiveness of these approaches depends on patient adherence to both types of interventions—behavioral and pharmacological [10]. Several randomized controlled trials (RCTs) have evaluated nonpharmacological interventions to reduce BP [5,6,11] and, in some studies, stimuli for lifestyle changes [12-14]. Text messaging interventions implemented in individuals with hypertension showed a small impact on BP compared to usual care [15]. In addition, similar levels of BP control have been observed with electronic monitoring and usual care [16]. Meaningful reductions in BP were observed with interventions involving frequent visits to a family doctor and adjustments of the therapeutic regimen [17], home BP monitoring [5], and home BP monitoring combined with medication titration, education, or lifestyle counseling [11]. Individuals with coronary heart disease undergoing an intervention based only on text messaging to improve lifestyle had a significant reduction of cardiovascular risk factors [18]; no clear-cut results were observed with an intervention based on text messages to improve medication adherence [19].

Innovative technologies can be used to achieve BP reduction, but it remains unknown if interventions should be focused only on hypertension control or should address lifestyle as well. Therefore, the purpose of this study is to compare the effectiveness of 3 strategies to reduce systolic BP assessed by ambulatory BP monitoring and improve lifestyle in comparison to the usual care of patients with hypertension (usual clinical treatment [UCT], control group). Our hypothesis is that participants assigned to active interventions will achieve greater BP reduction than those in the control group. The intervention by text messaging (TELEMEV) is a stimulus for adoption of a healthy lifestyle that may reduces BP, while telemonitoring of BP (TELEM) can enhance adherence to antihypertensive medication and stimulate healthy lifestyle. In addition, we hypothesized that participants who receive the combined intervention (TELEM+TELEMEV) will obtain greater reduction in BP than those submitted to individual interventions.

## Methods

### Study Design

**Figure 1:**
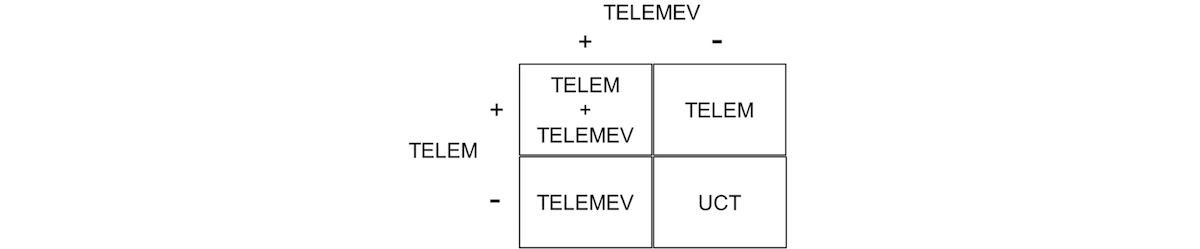
Factorial design of the trial showing the groups. TELEM: telemonitoring of blood pressure; TELEMEV: telemonitoring by text message; UCT: usual clinical treatment.

**Figure 2:**
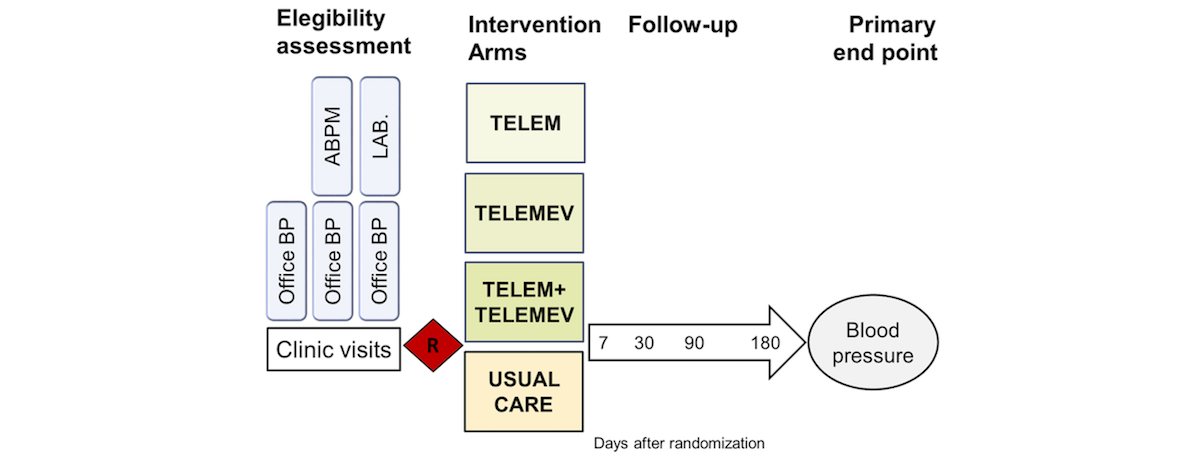
Overall design of the trial. ABPM: Ambulatory Blood Pressure Monitoring; BP: blood pressure; TELEM: telemonitoring of blood pressure; TELEM: telemonitoring by text message.

This is a factorial RCT (Figure 1) of effectiveness of the use of technologies—mobile phones and BP monitoring—to reduce systolic BP and change lifestyle. Figure 2 shows that participants are randomly allocated to 1 of 4 groups: telemonitoring BP (TELEM), telemonitoring messages (TELEMEV), telemonitoring BP plus telemonitoring messages (TELEM+TELEMEV), or UCT, with an allocation ratio of 1:1:2:1.

**Figure 3:**
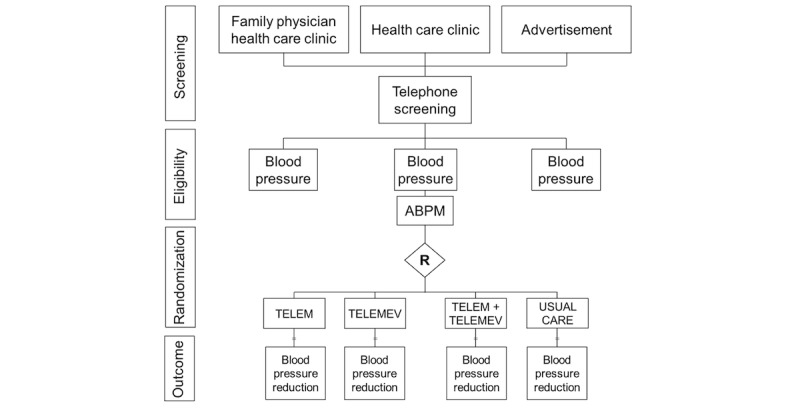
Study flowchart. usual clinical treatment. TELEM: telemonitoring of blood pressure; TELEMEV: telemonitoring by text message.

### Study Participants

Participants are recruited from primary care clinics face to face or by phone call to screen for eligibility or online advertisement. Figure 3 shows the screening of potential participants performed in 2 clinics, a Family Health Strategy and a Basic Health Care Unit. Eligible participants must be aged 30 to 75 years, have hypertension, be undergoing treatment with up to 2 medications for lowering BP, and possess a mobile phone with internet access.

The choice of antihypertensive medications is made prior to the trial at the discretion of the attending physician, and no titration of medications is made during the trial. Office standardized BP measurement is evaluated 4 times at the screening visit and 3 times in each eligibility visit. The first BP measurements are discarded, and an average systolic BP of ≥135 or diastolic BP of ≥85 mm Hg is required to be eligible, as well as systolic BP of ≥130 or diastolic BP ≥80 mm Hg in 24-hour ambulatory BP monitoring.

Participants with severe hypertension (systolic BP of ≥180 or diastolic BP of ≥110 mm Hg), life-threatening conditions, low life expectancy, other indications for antihypertensive medication, major cardiovascular event (acute myocardial infarction, stroke) in the last 6 months, diagnosis of secondary hypertension, participants from another RCT in the last 6 months, pregnant or lactating women, or those unable to understand the interventions are excluded. Data collection is performed at a clinical research center, Hospital de Clinicas de Porto Alegre, in southern Brazil. Automated office BP is also performed 6 times at each eligibility visit [20].

### Interventions

Participants in the 4 groups receive an illustrated booklet with recommendations for a healthy lifestyle and are required to adhere to the BP-lowering medications. In addition, participants who receive the active interventions are scheduled for an individual personalized session in which the booklet information is explained and specific recommendations are highlighted. The 4 arms are as follows:

- TELEM group: participants receive an automatic oscillometric device to measure BP 5 days a week and at least 1 day on the weekend. Participants are trained to use the monitor and instructed to perform 4 measurements per day (2 in the morning and 2 in the evening) using a standardized technique. Measurements are captured from the BP monitor by software developed for the study that also sends the BP measurements to the study coordination center. The software is adapted according to the participant's mobile phone brand and iOS or Android version. After BP measurements are sent to the data center, participants receive a prompt on the mobile phone with information about the value. At the end of the trial, participants will return the BP monitor.
- TELEMEV group: participants receive personalized, standardized text messages to stimulate lifestyle changes and adhere to BP-lowering medication. Messages focus on the adoption of dietary approaches to stop hypertension (DASH)-type diet, sodium restriction, reduction of alcohol intake, increase of physical activity, weight loss or control, and daily intake of BP-lowering medications. These messages are sent to mobile phones on 4 random business days at random business hours using software developed for the study. There is no contact other than the messages on the mobile phone. At the end of the trial, participants have no further access to the messages.
- TELEM-TELEMEV group: participants receive both interventions, telemonitoring of BP plus telemonitoring messages, as previously described.
- UCT: participants of the control group start the trial already on antihypertensive treatment, chosen at the discretion of the attending physician. At randomization, they receive a brief counseling about healthy lifestyle choices using the information in the booklet. Participants will not receive any technological tools to stimulate BP control or lifestyle modification.

### Outcomes and Their Assessment

Primary outcome is 24-hour systolic BP measured using ambulatory BP monitoring with the Ambulo ABP 2400 (Mortara Instrument) or Spacelabs 90207 (Spacelabs Healthcare) programmed to take measurements every 15 minutes from 0700 to 2300 hours and every 20 min from 2300 to 0700 hours. Office BP is assessed using an automatic oscillometric device (Omron HBP-1100 or HEM-705 CPN, Omron Healthcare Inc), and the average of 4 out of 6 measurements is used. Table 1 shows primary and secondary outcomes and their operational definitions.

### Randomization and Allocation Concealment

A computer-generated sequence was created in the random allocation software [22], which is used to randomly assign participants to 1 of 4 groups using permuted random block sizes of 4 and 8. The randomization sequence was generated prior to the trial initiation and is kept in the Research Electronic Data Capture software, which releases the allocated group only after completion of the enrollment. Just after completion of the enrollment of a participant, the randomized group is released, preventing the research team from anticipating to which arm the next participant will be allocated. Follow-up visits are scheduled for 7, 30, 90, and 180 days from randomization. At the 7-day visit, participants in the intervention groups can address any problems with image capture or text messages. The protocol was registered in the Plataforma Brasil (CAAE: 31423214000005327), a condition to be submitted to the Ethics Committee. It was approved by the Ethics Committee of the Hospital de Clinicas de Porto Alegre (GPPG number 16-0187), which is accredited by the Office of Human Research Protections as an institutional review board. After the institutional approval, the protocol was registered with ClinicalTrials.gov [NCT03005470]. A written informed consent is obtained from all participants according to the principles expressed in the Declaration of Helsinki. Potentially eligible participants will be evaluated in 3 consecutive clinical visits, held in the morning, in order to confirm eligibility criteria.

**Table 1:** Description of clinical and laboratory outcomes and their definitions.

Outcomes and definitions^a^		Primary outcome	Secondary outcomes
**Blood pressure**			
	Reduction in 24-hour systolic blood pressure in ambulatory blood pressure monitoring	X	
	Reduction in 24-hour diastolic blood pressure in ambulatory blood pressure monitoring		X
	Reduction in daytime systolic blood pressure		X
	Reduction in nighttime systolic blood pressure		X
	Office BP control (<130/80 mm Hg) [21]		X
**Sodium restriction**			
	Reduction in sodium urinary excretion (urinary spot)		X
**Healthy diet**			
	Increase in reported dietary intake (24-hour recall of food groups)		X
**Alcohol intake**			
	Reduction of reported intake (grams of ethanol per day)		X
**Physical activity**			
	Increase in average steps taken during 7 days (pedometer counting)		X
**Weight loss**			
	Reduction of at least 3 kg and average reduction		X

^a^Reduction or increase is calculated based on baseline and end of trial assessments.

**Table 2:** Sample size calculations based on a previous trial [5] and additional simulations maintaining constant 80% power and 95% confidence interval.

Intervention-to-control ratio	Average reduction in the intervention group, mm Hg (SD)	Average reduction in the control group, mm Hg (SD)	Calculated sample size per group	Total sample size
1:1:1:1	8.8 (13.1)	3.4 (11.6)	31	124
1:1:1:1	7.8 (13.1)	3.4 (11.6)	39	156
1:1:1:1	6.8 (13.1)	3.4 (11.6)	51	204
1:1:1:2	8.8 (13.1)	3.4 (11.6)	44/22	132
1:1:1:2	7.8 (13.1)	3.4 (11.6)	56/28	168
1:1:1:2	6.8 (13.1)	3.4 (11.6)	74/37	222

### Assessments During the Trial

The presence of risk factors associated with raised BP and cardiovascular risk are determined using standardized questionnaires in face-to-face interviews performed by staff members with undergraduate degrees in nutrition, biomedicine, or biology. Evaluations are performed at the beginning and end of the study using standardized interviews on prior morbidity, drug use, eating habits, and lifestyle. At the clinic, measurements are taken of weight; height; waist, hip, and neck circumferences; estimated body composition (bioelectrical impedance analysis); electrocardiography; retinography; and laboratory evaluation of cholesterol and fractions, triglycerides, fasting glucose, glycated hemoglobin A_1c_, creatinine, potassium, C-reactive protein, and urinary sodium. At home, measurements are taken of capillary glucose (before breakfast and dinner for 3 days; Accu-Chek glucose meter, Roche Diabetes Care Inc) and step count (Omron HJ-112 digital pocket pedometer, Omron Healthcare Inc) during the waking hours for 7 days. Participants have BP recorded in the office using an automatic oscillometric device and at home using a Spacelabs 90207 monitor (Spacelabs Healthcare).

In addition, participants are instructed not to change doses or type of antihypertensive medication during the trial.

### Sample Size Calculation and Statistical Analysis

The sample size calculation is based on results from a prior RCT with a factorial design [5]. Table 2 shows simulations for sample size calculations maintaining constant 80% power and 95% confidence interval. The largest sample size was obtained for a BP reduction of 6.8 [SD 13.1] mm Hg on 24-hour systolic BP in the intervention group compared to 3.4 [SD 11.6] mm Hg in the usual care treatment. Therefore, a sample of at least 222 participants is necessary to test our hypothesis.

Trial results will be analyzed using the intention-to-treat approach. The effectiveness of the active interventions will be tested in comparison to the control group. A pooled analysis of the differences between the active interventions versus control will be performed if there is no interaction. For continuous variables, the assumptions to use *t* tests will be verified using the Shapiro-Wilks test (for normal distribution) and the Levene test (for homogeneity of variance), and equal variances are assumed. Therefore, baseline characteristics will be analyzed using the *t* test for independent samples and chi-square test for categorical variables. Generalized estimating equations models will used to analyze the group, time, and time × group differences. The relative risk will be used to determine the corresponding relative risk reduction. *P*<.05 will be considered statistically significant, and .05<*P*<.15 will be considered a trend toward association.

## Results

This RCT was funded by two Brazilian agencies, and the results can be used to redefine public health policies. Data collection is ongoing and results are expected in early 2019. A 24-hour reduction in systolic BP of approximately 8.8 mm Hg is anticipated for participants in the electronic BP monitoring group with a similar effect in the text messaging group, against 3.4 mm Hg reduction in the usual care group. This reduction is clinically relevant and capable of impacting cardiovascular mortality. The two active interventions (TELEM and TELEMEV) are likely to provide equivalent benefits and, in this scenario, would be favorable to usual care in the public health system. Text messages seems to be more easily implemented at a lower cost.

## Discussion

### Lowering Blood Pressure

Hypertension is an inexorable and progressive condition that has deleterious effects on the heart, brain, and vascular system. Reducing BP and increasing control of hypertension are the main targets of interventions. However, if possible, the best intervention should be that which doesn’t require additional BP-lowering medications. Thus, any intervention capable of lowering BP or increasing BP control and, at the same time, attenuating other cardiovascular risk factors could represent an advantageous step in treatment.

This trial is the first to comprehensively compare two strategies for reducing BP and risk factors in cardiovascular disease. Results from previous trials have indicated the potential beneficial impact of self-monitoring programs [23], home blood pressure monitoring [5], and text messages [24], but these interventions have not been compared or assessed regarding other cardiovascular risk factors. The sample size, although larger than other trials, might be a limitation to test the secondary hypothesis.

### Conclusions

The evaluation of different health interventions allows us to select the most effective and lowest cost treatment to implement in clinical practice.

## Abbreviations

BP: blood pressure
CNPq: National Council for Scientific and Technological Development
RCT: randomized controlled trial
TELEM: telemonitoring of blood pressure
TELEMEV: telemonitoring by text message
UCT: usual clinical treatment
